# Laparoscopic surgery for massive ovarian edema during pregnancy: A case report

**DOI:** 10.1016/j.crwh.2021.e00318

**Published:** 2021-04-24

**Authors:** Shoko Saito, Megumi Yamamoto, Shizuha Iwaizumi, Hiroshi Yoshida, Hiroyuki Shigeta

**Affiliations:** a Department of Obstetrics and Gynecology, Yokohama Municipal Citizen's Hospital, 1-1 Mituzawa, Kanagawa-ku, Yokohama 221-0855, Japan; b Department of Obstetrics and Gynecology, Tokai University School of Medicine, 143 Shimokasuya, Isehara, Kanagawa 259-1193, Japan

**Keywords:** Massive ovarian edema, Pregnancy, Laparoscopic surgery

## Abstract

Massive ovarian edema (MOE) is a rare non-neoplastic clinicopathologic disease that is characterized by stromal edema and is caused by the partial or intermittent obstruction of venous and lymphatic drainage. The literature on MOE contains approximately 200 cases, but only 12 cases of MOE during pregnancy have been reported to date.

We report a case of MOE at 22 weeks of gestation that was diagnosed preoperatively, and the patient underwent laparoscopic surgery. Accurate preoperative diagnosis of MOE is important because it enables the selection of a therapeutic option, such as fundamental surgery, including adnexectomy; conservative surgery, including the release of torsion and ovarian biopsy; and conservative treatment without surgery.

MOE should be considered as a differential diagnosis for an enlarged ovary during pregnancy. Laparoscopic surgery may be a useful therapeutic option for MOE, especially during pregnancy.

## Highlights

• Massive ovarian edema is a rare non-neoplastic clinicopathologic disease.
• Massive ovarian edema should be considered as a differential diagnosis for an enlarged ovary during pregnancy.
• Laparoscopic surgery may be a useful therapeutic option for massive ovarian edema, especially during pregnancy.

## 1 Introduction

Massive ovarian edema (MOE) is a rare non-neoplastic clinicopathologic disease that is characterized by edema within the ovarian stroma and is caused by the obstruction of venous and lymphatic drainage. It usually occurs in young women, and common symptoms include lower abdominal pain, abdominal bloating, menstrual irregularity, and/or virilization. In many cases, it occurs unilaterally and is more common on the right side than on the left [1]. In most cases, surgery is performed. To date, approximately 200 cases of MOE have been reported. MOE during pregnancy was first reported by Gustafson in 1954 [2], and only 12 such cases have been reported in the literature. We report a case of MOE that occurred at 22 weeks of gestation and was diagnosed preoperatively. The patient underwent laparoscopic surgery. We obtained informed consent from the patient for the publication of this case report.

## 2 Case Presentation

A 23-year-old pregnant woman (gravida 1, para 0) attended hospital at 21 weeks of gestation with acute right lower abdominal pain. She had visited a clinic for prenatal checkups, and no adnexal mass was detected in the first trimester. The patient had no significant medical history. She had conceived spontaneously, and her prenatal course was uneventful.

**Fig. 1:**
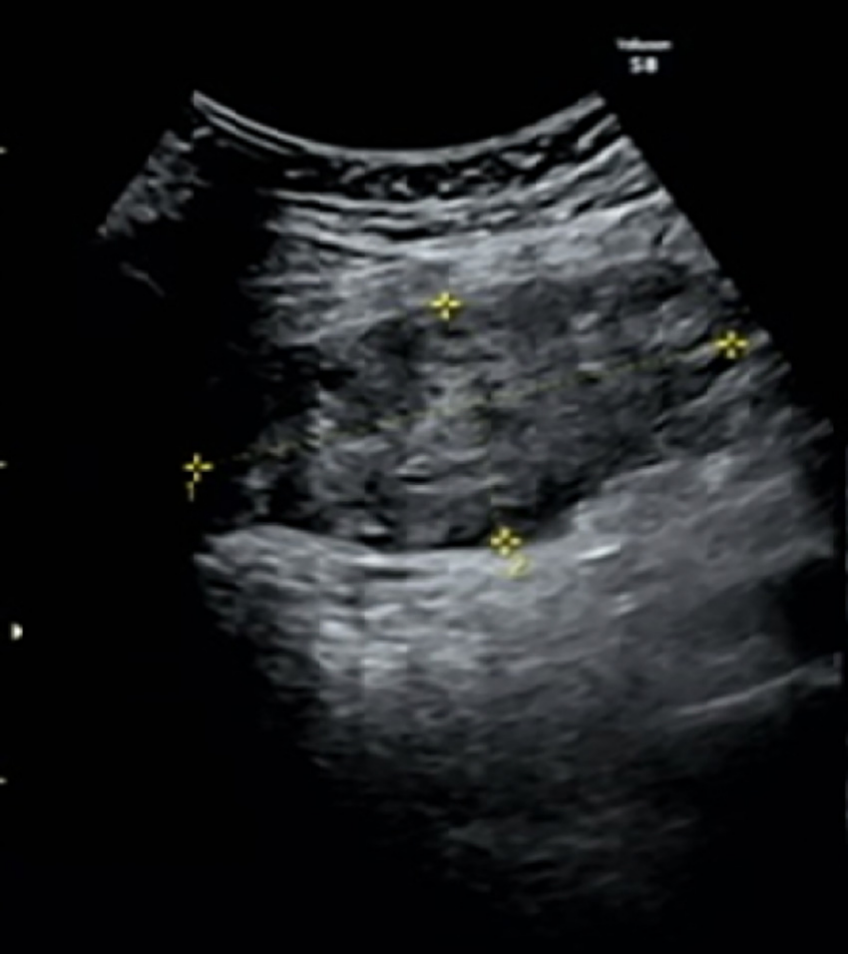
Transabdominal ultrasonography revealed a non-homogeneous low-echoic mass in the right adnexal region.

**Fig. 2:**
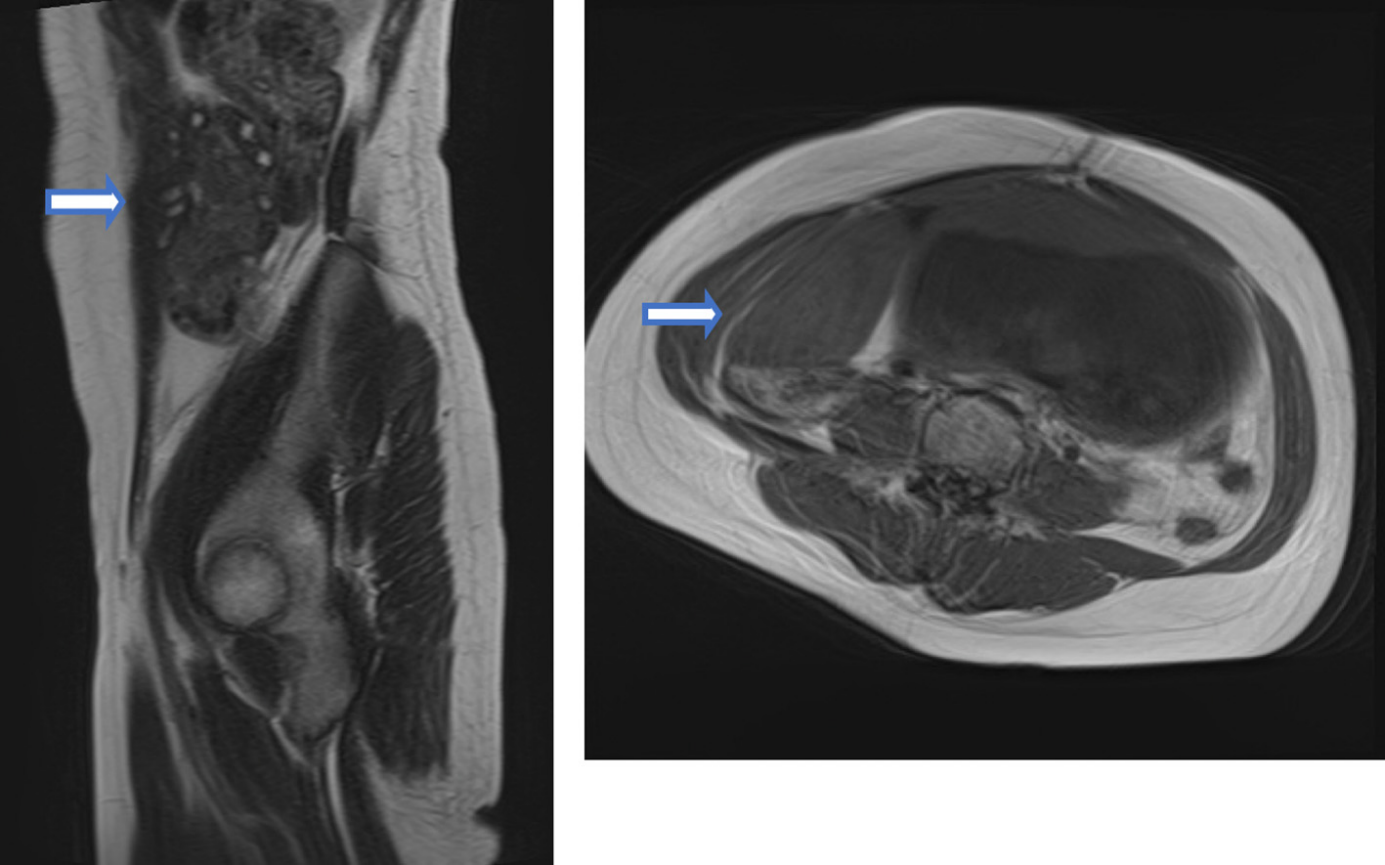
Magnetic resonance imaging (Left; T2-weighted image/sagittal, Right; T1-weighted image/axial). Multiple ovarian follicles were noted in the periphery of the mass on the T2-weighted image.

No obstetric abnormalities or adnexal masses were detected on transvaginal or transabdominal ultrasonography. The patient was admitted to hospital for observation. The abdominal pain initially improved but the pain flared up again after two days. Transabdominal ultrasonography revealed a non-homogeneous low-echoic mass (86 × 39 mm) in the right adnexal region (Fig. 1). On magnetic resonance imaging, the mass was suggestive of an enlarged ovary, with homogeneous hypointensity on T1-weighted images and isodensity to hyperintensity on T2-weighted images. Hyperintense multiple normal ovarian follicles were noted in the periphery of the mass on T2-weighted images (Fig. 2). The patient's serum lactate dehydrogenase level was within the normal range; the tumor marker was not measured. Based on these findings, MOE with torsion was strongly suspected, and the patient underwent laparoscopic surgery.

**Fig. 3:**
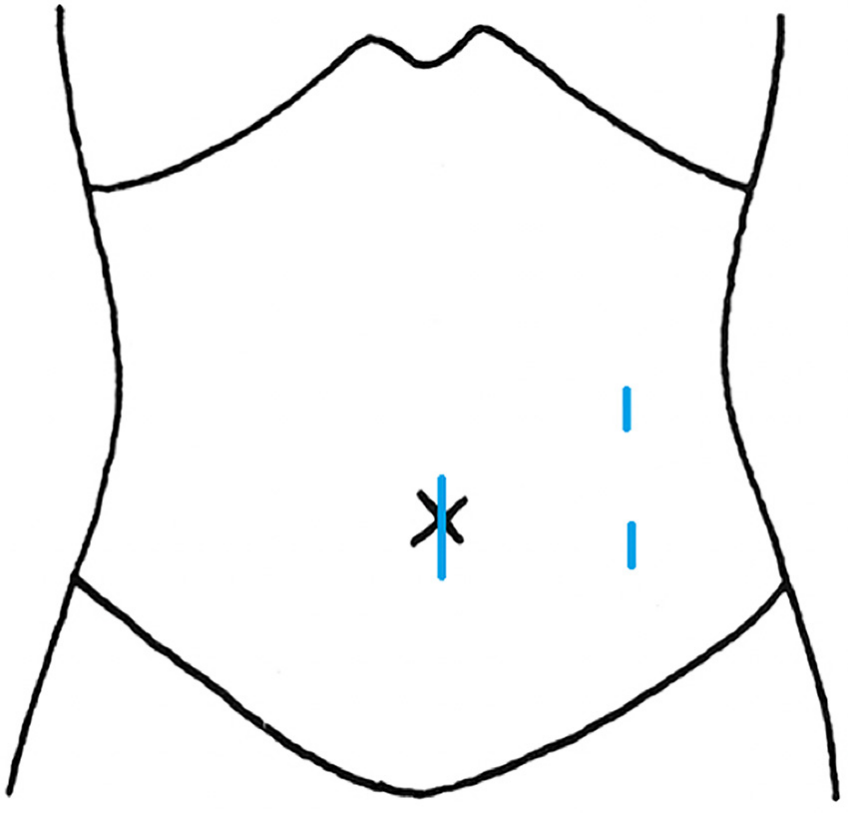
Trocar position. Two trocars were inserted through the left side to handle the right ovarian mass.

Intraoperatively, a 12-mm umbilical port was initially inserted with 10 mmHg of pneumoperitoneum pressure. Two 5-mm trocars were inserted through the left upper abdomen, at the umbilical level (Fig. 3). A right enlarged ovary with a swollen surface was observed. The ovarian mass appeared dark red; it had a diameter of 8 cm and was twisted by 720°. Because of its vulnerability, it was difficult to preserve the ovary. Therefore, we performed a right salpingo-oophorectomy. The infundibulopelvic ligament, ovarian ligament, and fallopian tube were divided using LigaSure^R^ (Medtronic, Tokyo, Japan). The divided right adnexa were removed through the umbilical port and kept in a pouch. The operation time was 47 min, and the blood loss was minimal.

**Fig. 4:**
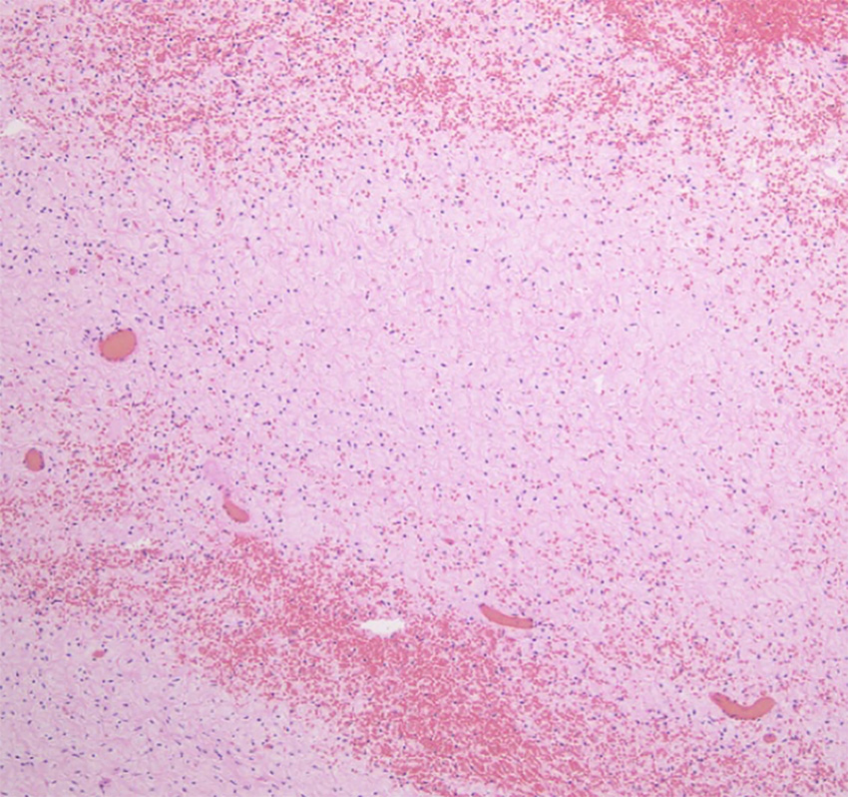
Microscopic finding (hematoxylin and eosin, ×100). Edematous stroma is shown.

Pathological examination of the excised mass revealed an edematous stroma with low cellularity and bleeding. No tumor-like region was observed (Fig. 4). The final diagnosis was MOE with torsion, and the patient was discharged 4 days after surgery with no complications. The course of her pregnancy was uneventful, and she had a normal vaginal delivery at 36 weeks of gestation.

## 3 Discussion

MOE is a rare non-neoplastic clinicopathologic disease that is characterized by stromal edema and is caused by the partial or intermittent obstruction of venous and lymphatic drainage. It is more common in young women than in elderly women, with a mean age of 20–22 years at diagnosis; however, postmenopausal cases have also been reported [3]. Most cases of MOE are unilateral and often present on the right side [1,[4], [5], [6], [7], [8]]. The sizes of the enlarged ovary ranges from 5 to 40 cm (mean: 10.4 cm) [1,5]. MOE can be complicated by torsion, as reported in 43–59% of surgically treated cases [3,9]. Ovarian enlargement is often associated with lower abdominal pain. Magnetic resonance imaging is useful for diagnosis. The enlarged ovaries are visualized as homogenous low-intensity areas on T1-weighted images and high-intensity areas on T2-weighted images, with follicles at the margin [1,10]. Serum lactate dehydrogenase levels and tumor markers are usually within the normal range [3]. In most cases, surgery is performed for removal of the mass [3]. Macroscopically, the surface of the mass is usually smooth and has a glossy white color, and the cracked surface is grayish-white and gelatinous [3]. Histologically, stromal, spindle-shaped cells are coarsely present with stromal edema [3,6]. Bleeding and necrosis often occur when torsion is observed.

The reported cases of MOE during pregnancy are summarized in Table 1 [1,2,[5], [6], [7], [8],[10], [11], [12], [13]]. Including our case of MOE during pregnancy, the median size of the enlarged ovary in reported cases is 10 cm (range: 5–28 cm), and in 71.4% of the cases the lesions are on the right. The patients were diagnosed between 11 and 32 weeks of gestation. In terms of treatment, surgery was performed in 9 cases, and conservative treatment was continued in 3 cases; the treatment modality was not described in 1 case.

**Table 1:** Reports of massive ovarian edema during pregnancy.

Author [reference no.]	Age	Gestational week	Symptom	Site of ovarian edema	Size (cm)	Torsion	Treatment	Pregnancy outcome
Gustafson GW [2]	22	24	Abdominal pain	Left	9	ND	Oophorectomy	Stillbirth at 24 weeks
Chervenak FA [5]	19	32	Abdominal pain	Right	28 × 26 × 9	Yes	Right oophorectomy, Left biopsy	Vaginal delivery at 32 weeks (2 days after surgery)
Young RH [13]	16	First trimester	Abdominal pain	Right	8	Yes	Oophorectomy	ND
Weinreb JC [12]	ND	11	Abdominal pain	Right	5 × 3.5	Yes	ND	ND
Lambert B [7]	26	1; 12 2; 27	Abdominal pain	1; Right 2; Left	1; 12 × 15 2; 11 × 5 × 2	1; Yes 2; No	1; Oophorectomy 2; Partial resection after delivery	Delivery at 33 weeks
Hall BP [6]	21	17	None	Right	10 × 8.5 × 5	Partially	Oophorectomy	ND
Schmidt P [11]	28	16	None	Right	11.5	No	Oophorectomy	Normal delivery
Kawaguchi R [8]	40	12	Abdominal pain	Left	9 × 6	Partially	Oophorectomy	Normal delivery at 39 weeks
Coakley FV [10]	40	26	None	Right	ND	No	Conservative (no surgery)	Normal delivery
	31	27	Abdominal pain	Right	ND	No	Conservative (no surgery)	Cesarean section at 42 weeks
	36	31	Abdominal pain	Right	ND	ND	Conservative (no surgery)	Normal delivery at 39 weeks
Gobara A [1]	24	12	Abdominal pain	Left	9.9 × 6.1 × 6	No	Partial resection	Normal delivery
Present case	23	22	Abdominal pain	Right	8.6 × 3.9	Yes	Oophorectomy	Vaginal delivery at 36 weeks

ND; not described.

In MOE occurring during pregnancy, the frequency of torsion is approximately half, which is almost the same as in non-pregnant women. It has been reported that MOE is caused by intravascular congestion resulting from the compression of the ovary between the pregnant uterus and the adjacent structures, such as the pelvic wall [1]. There is another report of MOE in a woman with ovulation-induced pregnancy, possibly due to ovulation-induced ovarian swelling [8]. The pathophysiology of MOE in the case reported here was unclear. However, it is speculated that intravascular congestion, caused perhaps by partial or intermittent torsion, resulted in lower abdominal pain and finally led to MOE.

The preoperative diagnosis of MOE is important, especially during pregnancy. There are three therapeutic options: fundamental surgery, including adnexectomy; conservative surgery, including the release of torsion and ovarian biopsy [3]; and conservative treatment without surgery. Choice of option depends on feasibility. If a malignant tumor cannot be ruled out, adnexectomy via laparotomy is essential.

There are no reports of preserving the ovary after releasing torsion in cases of MOE with torsion during pregnancy; however, it may be possible to preserve the ovary because it has been reported that ovarian function is maintained even if the ovarian color does not improve after the torsion is surgically released [14]. In cases of MOE without torsion, conservative treatment has been reported to be safe, and normalization of ovarian function has been confirmed after delivery [10].

During pregnancy, laparotomy results in a larger incision because of the enlarged uterus. However, in laparoscopic surgery, it is possible to obtain a good surgical field by selecting appropriate ports, considering the gravid uterus. Laparoscopic surgery is useful and safe for ovarian tumors during pregnancy [15,16]. Therefore, laparoscopic surgery may be useful therapeutic option for MOE during pregnancy. In the present case, surgery was performed with 10 mmHg CO_2_ pressure because a CO_2_ insufflation of 10–15 mmHg can be safely used during laparoscopy in pregnant patients [16].

## 4 Conclusion

MOE should be considered as a differential diagnosis for ovarian tumors during pregnancy. Considering ovarian preservation, MOE should be diagnosed preoperatively. Laparoscopic surgery may be a useful therapeutic option for MOE during pregnancy.
